# Long-Term Dietary Changes after Diagnosis of Rheumatoid Arthritis in Swedish Women: Data from a Population-Based Cohort

**DOI:** 10.1155/2018/9152480

**Published:** 2018-06-11

**Authors:** Cecilia Lourdudoss, Laurent Arnaud, Alicja Wolk, Ronald F. van Vollenhoven, Daniela Di Giuseppe

**Affiliations:** ^1^ClinTRID, D1:00, Karolinska University Hospital, Solna, 17176 Stockholm, Sweden; ^2^Institute of Environmental Medicine (IMM), C6, Nutritional Epidemiology, Box 210, 17177 Stockholm, Sweden; ^3^Eugeniahemmet, T2, Karolinska Universitetssjukhuset, Solna, 17176 Stockholm, Sweden

## Abstract

**Objective:**

To investigate long-term dietary changes after rheumatoid arthritis (RA) diagnosis in Swedish women, compared to women without RA.

**Methods:**

This study included 21,602 women from the Swedish Mammography Cohort (SMC), who completed dietary questionnaires in 1997 and 2009. Between 1997 and 2009, 191 women were diagnosed with RA. Dietary changes after RA diagnosis were analyzed based on intake of 82 food items. Statistical analysis included linear mixed models.

**Results:**

Women with RA, compared to women without RA, had significantly lower intake (mean servings per week) of animal products such as black pudding, egg, kidney, and liver paste (2.94±2.73 versus 2.45±1.82, p=0.010) and dairy products (35.14±20.02 versus 28.42±16.10, p=0.040) in 1997 and of cereals and grains (31.01±15.54 versus 28.00±14.98, p=0.009) in 2009. However, multivariable adjusted changes in dietary intake from 1997 to 2009 did not show any significant difference in intake. Nevertheless, women without RA increased their intake of whole wheat bread, wheat/oat bran, and rice more than women with RA.

**Conclusion:**

Women who had been diagnosed with RA had similar dietary patterns over time as the general population; these women did not remarkably change their diet over time due to their disease. Dietary recommendations for RA patients are needed.

## 1. Introduction

Rheumatoid arthritis (RA) is a chronic, systemic autoimmune disease with unknown cause. RA affects mostly joints but may also affect other tissues and organs. Destruction of articular cartilage and loss of function of the joints are often results of the pathology of the disease [1]. RA is two to three times more common in women than men and the estimated global prevalence is 0.5-1% [2].

The interest in association of diet with RA incidence and progression has increased among researchers and RA patients over the last decade. However, there are relatively low levels of evidence of dietary impact on the course of RA. Certain specific diets, such as vegetarian, Mediterranean, vegan, and gluten-free diet, have been shown to ameliorate to some extent the disease status of RA [3–8]. The Mediterranean diet encourages increased intake of fruits, vegetables, legumes, fatty fish, and olive oil and reduced/moderate intake of red meat and dairy products [9]. Increased fruit and vegetable intake may reduce the risk of RA [10–12] and may also be beneficial for prevalent RA patients [8, 13]. High consumption of fatty fish containing omega-3 fatty acids may reduce the inflammation compared to high consumption of red meat. Omega-3 fatty acids from either diet or supplements have been associated with decreased disease activity in RA through anti-inflammatory mechanisms [3, 14–16]. Higher dietary intake of omega-3 fatty acids has been associated with decreased serum levels of tumor necrosis factor and C-reactive protein in RA [17].

Although, several interventions of dietary aspects have shown benefits for RA, there are no studies on change in diet among women with RA due to their disease. We hypothesized that women who have been diagnosed with RA change their diet in order to improve their disease condition. The aim of this study was to investigate the long-term changes in diet in women after RA diagnosis compared to women without RA from the general Swedish population.

## 2. Materials and Methods

### 2.1. Study Population

This study included 21,602 women from the Swedish Mammography Cohort (SMC), a large population-based cohort from central Sweden. The study design of the SMC has been previously described in detail [18]. The SMC was established in 1987-1990 and included women born from 1914 to 1948 and residing in Uppsala and Västmanland counties. Women were asked to complete a self-administered questionnaire regarding food intake as well as other lifestyle factors. The questionnaires were distributed at baseline in 1990 and at two follow-ups in 1997 and in 2009. Our study was based on food frequency questionnaires (FFQ) from 1997 and 2009.

The Swedish Rheumatology Quality (SRQ) Register is a nationwide ongoing registry of incident rheumatic patients. The SMC was linked to both the SRQ and the Patient Register at the Swedish National Board of Health and Welfare in order to track incident RA cases from 1997 to 2009. The Patient Register includes the Outpatient Register, which contains information on all the outpatient specialist visits from 2001, including visits from both public and private caregivers, and the Inpatient Register, which contains information on all hospitalizations from 1987. During the period from 1997 to 2009, 191 women were diagnosed with RA within the SMC. Thus, this study included 191 women with RA and 21,411 women without RA in the study period.

### 2.2. Dietary Assessment

Dietary assessment was based on validated FFQ, which included questions regarding participants' frequency intake of several food items and beverages during the previous year from assessment dates in 1997 and 2009. The FFQ included 96 and 132 food items in 1997 and 2009, respectively. Food frequency intake ranged over eight categories from* never* to* ≥3 times per day*. Portion size (i.e., small, median, large) and quantity (i.e., slice, cup, glass, deciliter) of frequently consumed food items in Sweden were collected. Partial nonresponse was considered as zero intake [19]. This study focused on 82 common food items that were included in both FFQs; all the food items were categorized into thirteen categories (the appendix).

### 2.3. Statistical Analysis

Baseline characteristics from 1997 and dietary intake from 1997 and 2009 were compared between women with and without a diagnosis of RA with Mann–Whitney* U* test for continuous variables (mean and standard deviation (SD)) as well as Pearson's chi-square test for proportions (%). Dietary changes after RA diagnosis were analyzed based on the food frequency intake in 1997 and 2009 with linear mixed models (IBM SPSS Statistics 23).

Linear mixed models for repeated measures were used in order to compare the dietary mean intake per week in 1997 and 2009. Separate models were built for each outcome of interest (food category/item). Each of the food categories/items was separately included as the dependent variable in the model, with 1997 and 2009 as fixed effects. These analyses were performed separately in women with and without RA. In addition, linear mixed models were performed in order to investigate if the changes in dietary intake from 1997 to 2009 differed between women with and without RA. All linear mixed models were adjusted for age, smoking, body mass index (BMI), and alcohol intake in 1997. Age (years), BMI (Kg/m^2^), and alcohol intake (g/day) were modeled as quartiles, while smoking status was categorized into never, former, and current smoker.

### 2.4. Ethical Standards

This study was approved by the Regional Ethical Review Board at Karolinska Institutet, Stockholm, Sweden (reference number: 2013/1019-31/1), and has therefore been performed in accordance with the ethical standards laid down in the 1964 Declaration of Helsinki and its later amendments.

## 3. Results

### 3.1. Characteristics of Study Participants

This study included 191 women with RA and 21,411 women without RA between 1997 and 2009. Baseline characteristics of the study participants are presented in Table 1. Age, BMI, alcohol intake, and education level did not differ between women with and without RA; however, smoking was more common among women with RA.

### 3.2. Dietary Intake in 1997 and 2009

Women with RA, compared to women without RA, had significantly lower intake of ‘other animal products' (black pudding, egg, kidney, and liver paste) and dairy products in 1997 and cereals and grains in 2009 (Table 2). When comparing separately the intake of the 82 food items, women with RA had significantly lower intake of apple/pear, wheat/oat bran, and black pudding in 1997 and low-fat milk, fried potatoes, broccoli/Brussels sprouts, lean fish, and nuts/almonds in 2009 (results not shown).

### 3.3. Changes in Dietary Intake between 1997 and 2009

Results from mixed models showed that the food frequency intake of 8 (61.5%) food categories changed statistically significantly from 1997 to 2009* within *women with RA and 13 (100%) food categories within women without RA. The changes of intake (increase/decrease) of each food category during the study period were the same in both groups (results not shown).

Multivariable adjusted dietary changes of all the food categories* between *women with and without RA are presented in Table 3. Changes in dietary intake from 1997 to 2009 did not differ significantly between women with and without RA for all the food categories. Similar trends were seen when looking at all the food items separately. The very few significant differences were intake of whole wheat bread, wheat/oat bran, and rice. The increase in intake of these three food items among women without RA was higher than among RA patients. Based on these results, we further analyzed whole grains (crispbread, whole wheat bread, oat meal, gruel, cereals/muesli) and refined grains (white bread/loaf, pasta, rice, pancakes, biscuit/wafer, buns/cakes), respectively. There were no statistically significant differences in the intake of different types of grains during the study period between women with and without RA.

## 4. Discussion

This study investigated the long-term changes in diet after RA diagnosis. The main results indicated that women who had been diagnosed with RA had similar dietary habits over time as the general population, and these women did not remarkably change their diet due to their disease.

Results from the multivariable adjusted linear mixed model showed no significant difference in the changes of dietary intake based on food categories. However, when looking more specifically into each food item separately, the results showed that women without RA increased their intake of whole wheat bread, rice, and wheat/oat bran more than women with RA. Based on these findings, we speculated that some of the women with RA may have been treated with glucocorticoids and therefore had increased appetite, which may have increased their intake of refined grains rather than whole grains, compared to women without RA. However, further analysis did not show any difference regarding different types of grains between women with and without RA.

Healthy food and eating habits have been highlighted in the media during the last decade. The interest in these topics may also have increased in patients with RA. Although the information and the access to healthy eating have been increased, it still remains a struggle to sustain new dietary changes over time [20]. Many previous studies have shown beneficial effect of diet changing after RA diagnosis [4, 8, 11, 21–30], but the evidence did not lead to specific dietary recommendation for RA patients.

The majority of our results did not show significant changes in diet between 1997 and 2009. A nonadjusted comparison of means showed a lower intake among women with RA compared to women without RA of apple/pear, wheat/oat bran, and black pudding in 1997 and of low-fat milk, fried potatoes, broccoli/Brussels sprouts, lean fish, nuts, and almonds in 2009.

This study is, to our knowledge, the first to assess the long-term dietary changes after RA diagnosis in a large population-based cohort. The SMC has a prospective design including a large population and the use of validated FFQs. The participants of the SMC were representative of elderly women from the general population in 2009; however, the younger generation may have been more prone to change their diet after RA diagnosis, as hunger, appetite, and food intake get affected with age [31]. Dietary data from FFQ were based on self-reported dietary consumption; therefore under- and overreporting may have occurred when completing the FFQ in 2009. Nevertheless, under- and overreporting were taken into account during the validation of the FFQs. Long-term changes in diet were based on dietary assessment from only two time points with twelve years apart; therefore, any important short-term changes subsequent to RA diagnosis may have been neglected. Because of the high number of tests performed when evaluating the effect of the 82 food items separately, reported statistically significant associations could be chance findings. Clinical aspects of RA were not considered due to large proportion of missing data (~70%).

## 5. Conclusions

This study revealed that women who had been diagnosed with RA had similar dietary patterns over time as the general population and that these women did not remarkably change their diet due to their disease. Improved nutritious diet may have possible advantages, and specific dietary recommendations for patients with RA are needed.

## Figures and Tables

**Table 1:** Baseline characteristics of participants of the Swedish Mammography Cohort with or without RA diagnosis between 1997 and 2009.

**Characteristics** **(1997)**	**RA** **(n=191)**	**No RA** **(n=21,411)**	**p value** ^**1**^
Age (yrs), mean ± SD	59 ± 7	59 ± 8	N.S.
BMI (Kg/m^2^), mean ± SD	24.5 ± 3.6	25.0 ± 3.8	N.S.
Alcohol (g/day), mean ± SD	4.9 ± 5.2	4.8 ±5.2	N.S.
Smoking, %			<0.001
(i) Never	33.9	52.8	
(ii) Former	31.7	25.5	
(iii) Current	34.4	21.7	
Education, %			N.S.
(i) Less than high school	33.0	34.8	
(ii) High school	46.1	41.6	
(iii) University	20.9	23.6	

BMI: body mass index; N.S.: not significant; RA: rheumatoid arthritis; SD: standard deviation.

^1^The significance level is 0.05.

**Table 2:** Dietary mean intake among women with RA and without RA in 1997 and 2009.

		**Intake, servings per week (mean ± SD)**		
**Year**	**Food category ** ^**1**^	**RA (n=191)**	**No RA (n=21,411)**	**p value** ^**2**^
**1997**	Fruits	14.00 ±8.61	15.12 ± 9.80	N.S.
	Vegetables	29.26 ± 14.49	30.10 ± 15.47	N.S.
	Cereals and grains	34.79 ± 12.74	36.68 ± 15.47	N.S.
	White meat	0.77 ± 0.91	0.70 ± 0.77	N.S.
	Red meat	5.81 ± 3.57	6.30 ± 3.99	N.S.
	Fish and seafood	3.92 ± 2.87	3.71 ± 2.80	N.S.
	**Other animal products**	**2.45 ± 1.82**	**2.94 ± 2.73**	**0.010**
	**Dairy products**	**28.42 ± 16.10**	**35.14 ± 20.02**	**0.040**
	Alcohol	4.90 ± 4.27	5.04 ± 4.83	N.S.
	Coffee and tea	27.79 ± 11.62	27.23 ± 12.88	N.S.
	High sugary products	28.00 ± 24.78	24.57 ± 19.11	N.S.
	Nuts, salty snacks/foods	1.26 ± 0.91	1.33 ± 1.47	N.S.
	Sauce	2.31 ± 2.10	2.24 ± 2.24	N.S.

**2009**	Fruits	13.86 ± 8.47	14.98 ± 9.73	N.S.
	Vegetables	26.60 ± 12.60	27.51 ± 13.30	N.S.
	**Cereals and grains**	**28.00 ± 14.98**	**31.01 ± 15.54**	**0.009**
	White meat	0.98 ± 0.77	1.05 ± 0.84	N.S.
	Red meat	6.86 ± 4.34	7.56 ± 4.97	N.S.
	Fish and seafood	4.20 ± 2.59	4.48 ± 2.87	N.S.
	Other animal products	5.18 ± 6.65	5.53 ± 6.72	N.S.
	Dairy products	37.52 ± 23.59	35.77 ± 21.56	N.S.
	Alcohol	2.03 ± 2.03	2.45 ± 2.52	N.S.
	Coffee and tea	25.06 ± 14.70	25.06 ± 14.00	N.S.
	High sugary products	18.27 ± 15.26	16.73 ± 15.19	N.S.
	Nuts, salty snacks/foods	1.33 ± 1.47	1.75 ± 2.45	N.S.
	Sauce	1.75 ±1.68	1.89 ± 2.24	N.S.

N.S.: not significant; RA: rheumatoid arthritis; SD: standard deviation.

^1^Food categorization is presented in the appendix.

^2^p value from Mann–Whitney *U* test; the significance level is 0.05.

**Table 3:** Multivariable adjusted changes in dietary intake (servings/week) from 1997 and 2009 *between* women with and without RA.

	**Servings per week 1997→2009 (mean ± SD)** ^**2**^		
**Food category** ^**1**^	**RA (n=191)**	**No RA (n=21,411)**	**p value** ^**3**^
Fruits	14.08 ± 0.58	14.45 ± 0.06	N.S.
Vegetables	26.75 ± 0.88	27.74 ± 0.08	N.S.
Cereals and grains	31.12 ± 0.89	32.19 ± 0.08	N.S.
White meat	0.80 ± 0.04	0.80 ± 0.01	N.S.
Red meat	6.15 ± 0.25	6.33 ± 0.02	N.S.
Fish and seafood	3.86 ± 0.16	3.87 ± 0.01	N.S.
Other animal products	2.69 ± 0.18	3.02 ± 0.01	N.S.
Dairy products	33.34 ± 1.14	32.49 ± 0.11	N.S.
Alcohol	2.31 ± 0.14	2.46 ± 0.01	N.S.
Coffee and tea	22.44 ± 0.80	22.82 ± 0.01	N.S.
High sugary products	18.20 ± 0.90	17.19 ± 0.08	N.S.
Nuts, salty snacks/foods	1.09 ± 0.08	1.26 ± 0.01	N.S.
Sauce	1.77 ± 0.13	1.87 ± 0.01	N.S.

N.S.: not significant; RA: rheumatoid arthritis; SD: standard deviation.

^1^Food categorization is presented in the appendix.

^2^Adjustment for age (quartiles), smoking (never, former, current), BMI (quartiles), and alcohol intake (quartiles) in 1997.

^3^p value from linear mixed model; the significance level is 0.05.

**Table 4:** Food categorization.

**Food category**	**Food item**
Fruits	Apple
	Banana
	Berry
	Orange
	Orange juice
	Other fruits

Vegetables	Beetroot
	Broccoli
	Cabbage
	Carrot
	Cauliflower
	Cooked potatoes
	Garlic
	Lettuce
	Mixed vegetables
	Onion
	Pea soap
	Peas
	Pepper
	Roast potatoes
	Spinach
	Tomato

Cereals and grains	Flakes
	Gruel
	Hard bread
	Oatmeal
	Pancakes
	Rice
	Spaghetti
	Wheat bran
	White bread
	Whole meal bread

White meat	Chicken

Red meat	Ham
	Meatballs
	Pork
	Sausage
	Veal

Fish and seafood	Caviar
	Codfish
	Herring
	Salmon
	Shellfish

Other animal products	Black pudding
	Egg
	Kidney
	Liver paste

Dairy products	Cheese (<17%)
	Cheese (>24%)
	Cottage cheese
	Cream
	Crème fraiche
	Milk (0.5%)
	Milk (1.5%)
	Milk (3.0%)
	Sour milk, yogurt (0.5%)
	Sour milk, yogurt (3.0%)

Alcohol	Beer (2.8%)
	Light beer (0.5%)
	Spirit/hard liquor (40%)
	Strong beer (4.5%)
	Strong wine
	Wine (red, white)

Coffee and tea	Coffee
	Tea (black, green, red)

High sugary products	Biscuit
	Buns and cakes
	Cake
	Candy
	Chocolate
	Ice cream
	Jam
	Soft drink/soda
	Stewed fruit puree
	Sugar

Nuts, salty snacks/foods	Chips
	Nuts
	Pizza
	Potato crisps

Sauce	Ketchup
	Mayonnaise
	Salad dressing

## Data Availability

The data used to support the findings of this study were provided by the Institute of Environmental Medicine at Karolinska Institutet, Stockholm, Sweden, under license and therefore cannot be freely available.

## Appendix

See Table 4.
